# Bilaterale Tieftonschwerhörigkeit und Tinnitus nach Spinalanästhesie bei Sectio caesarea

**DOI:** 10.1007/s00106-026-01781-x

**Published:** 2026-06-18

**Authors:** Cosima C. Hoch, Christian Haslinger, Francesco Capecchi, Sabrina Beham, Dorothe Veraguth, David Bächinger

**Affiliations:** 1 Klinik für Ohren‑, Nasen‑, Hals- und Gesichtschirurgie, Universitätsspital Zürich, Frauenklinikstr. 24, 8091 Zürich, Schweiz; 2 Universität Zürich, Zürich, Schweiz; 3 Klinik für Geburtshilfe, Universitätsspital Zürich, Zürich, Schweiz; 4 Klinik für Neurologie, Universitätsspital Zürich, Zürich, Schweiz; 5 Institut für Anästhesiologie und Perioperative Medizin, Universitätsspital Zürich, Zürich, Schweiz

## Anamnese

Eine 39-jährige Patientin wurde zwei Tage nach geplanter Sectio caesarea (39. Schwangerschaftswoche) in Spinalanästhesie konsiliarisch in der Hals-Nasen-Ohren-Klinik vorgestellt. Seit der Spinalanästhesie berichtete sie über eine neu aufgetretene beidseitige Schwerhörigkeit mit dumpfer, im Tieftonbereich veränderter Hörwahrnehmung, ein persistierendes, zentral wahrgenommenes rauschendes Ohrgeräusch und eine Autophonie. Begleitend bestanden okzipitale Kopfschmerzen (maximal 8/10 auf der visuellen Analogskala) ohne eindeutige Lageabhängigkeit und ein intermittierender ungerichteter Schwindel. Otalgie und Otorrhoe wurden verneint. Otologische Vorerkrankungen, Allergien und Noxen lagen nicht vor.

## Klinischer Befund

Die Patientin befand sich in gutem Allgemein- und Ernährungszustand, war afebril und kreislaufstabil. Die ohrmikroskopische Untersuchung ergab beidseits unauffällige Trommelfellbefunde. In den Stimmgabelversuchen zeigte sich ein mittiger Weber-Versuch bei beidseits positivem Rinne-Versuch.

In der klinisch-neurootologischen Untersuchung fand sich weder ein Spontan- noch ein Provokationsnystagmus. Der klinische Kopfimpulstest und die Okulomotorik waren unauffällig, ebenso der Romberg-Test. Die orientierende neurologische Untersuchung ergab keine fokalneurologischen Defizite.

## Audiologische Diagnostik

**Abb. 1:**
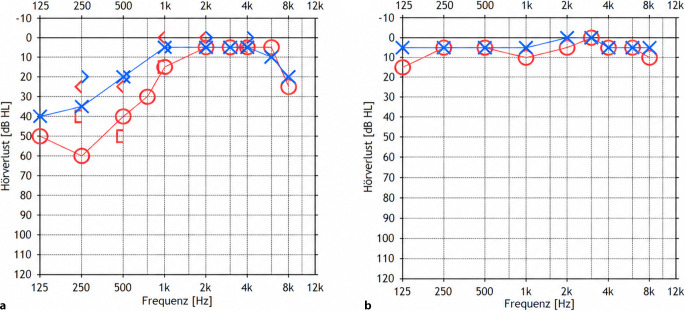
Reintonaudiogramme im Verlauf (*dB HL* „decibel hearing level“): **a** initial nachgewiesene beidseitige tieftonbetonte sensorineurale Schwerhörigkeit, **b** Verlaufsreintonaudiogramm nach einer Woche mit vollständiger Rückbildung der Schwerhörigkeit im Sinne einer Normakusis beidseits

Im Reintonaudiogramm zeigte sich eine beidseitige, rechts- sowie tieftonbetonte sensorineurale Schwerhörigkeit (Abb. 1a).

## Wie lautet Ihre Diagnose?

**Diagnose:** Postpunktionelles Liquorverlustsyndrom nach Spinalanästhesie mit bilateraler Tieftonschwerhörigkeit und Tinnitus

## Therapie und Verlauf

Bei typischen Ohrsymptomen unmittelbar nach Spinalanästhesie mit begleitenden okzipitalen Kopfschmerzen wurde die Verdachtsdiagnose eines postpunktionellen Liquorverlustsyndroms (syn.: postpunktionelles Syndrom [PPS]) gestellt. Es wurde ein konservatives Vorgehen mit körperlicher Schonung, ausreichender Hydrierung, gesteigerter Koffeinzufuhr sowie analgetischer Therapie mit Paracetamol und Koffein eingeleitet.

Im Verlauf persistierten die Schwerhörigkeit, der Tinnitus sowie die Autophonie zunächst über die ersten Tage mit fluktuierender Symptomatik. Die Kopfschmerzen und der Schwindel bildeten sich innerhalb von fünf Tagen vollständig zurück, während die Gehörveränderungen länger bestanden.

In der Verlaufskontrolle nach einer Woche zeigte sich eine komplette Regredienz der Schwerhörigkeit bei lediglich noch diskretem Tinnitus. Im Verlaufsreintonaudiogramm fand sich eine beidseitige Normakusis (Abb. 1b). Bei der Verlaufskontrolle nach sechs Wochen berichtete die Patientin über einen weiterhin bestehenden, jedoch deutlich reduzierten Tinnitus bei subjektiv normalem Hörvermögen.

Ein epiduraler Blutpatch (EBP) wurde als mögliche Therapieeskalation erwogen, war jedoch aufgrund der raschen spontanen Besserung nicht erforderlich. Beim EBP wird autologes Blut in den Epiduralraum injiziert, um den duralen Defekt abzudichten, den Liquorverlust zu reduzieren und dadurch die Symptome des PPS zu lindern. Die Patientin wurde über das Risiko persistierender cochleovestibulärer Beschwerden, insbesondere eines fortbestehenden Tinnitus und einer persistierenden Hörminderung, aufgeklärt. Eine audiometrische Verlaufskontrolle nach drei Monaten wurde vereinbart.

## Diskussion

Das PPS ist eine bekannte Komplikation nach Spinalanästhesie und geht typischerweise mit orthostatischen Kopfschmerzen einher [1]. Gehörveränderungen, insbesondere tieftonbetonte sensorineurale Schwerhörigkeit, Tinnitus, Autophonie oder Schwindel, stellen eine seltene Komplikation dar, die von den Patientinnen und Patienten meist bemerkt, im perioperativen Verlauf jedoch nicht immer systematisch erfragt oder unmittelbar mit der vorausgegangenen Spinalanästhesie in Zusammenhang gebracht wird [2, 3]. Die berichtete Häufigkeit variiert erheblich und hängt unter anderem von patientenbezogenen und technischen Faktoren ab [1].

Bemerkenswert ist im vorliegenden Fall die atypische Präsentation: Die Kopfschmerzen waren nicht klar lageabhängig und bildeten sich rasch zurück, während die Ohrsymptome die führende und am längsten persistierende Manifestation darstellten. Dies unterstreicht die Bedeutung, bei neu aufgetretener tieftonbetonter Hörstörung gezielt nach einer vorausgegangenen Spinalanästhesie zu fragen, auch wenn klassische orthostatische Kopfschmerzen fehlen.

Differenzialdiagnostisch ist bei akuter bilateraler tieftonbetonter sensorineuraler Schwerhörigkeit insbesondere eine immunvermittelte Innenohrerkrankung (syn.: autoimmune Innenohrerkrankung) abzugrenzen, die eine immunsuppressive Therapie erfordern würde. Im vorliegenden Fall sprachen der enge zeitliche Zusammenhang mit der Spinalanästhesie, die Begleitsymptome und die spontane Regredienz gegen diese Diagnose. Weitere Differenzialdiagnosen wie ein bilateraler (idiopathischer) Hörsturz, ein Morbus Menière oder eine Perilymphfistel, welche eine Therapie mit Kortikosteroiden nach sich ziehen würden, waren aufgrund der Bilateralität, der Begleitsymptome bzw. des typischen Auslösers und des Verlaufs unwahrscheinlich.

Die Patientin wies zudem mehrere etablierte Risikofaktoren für ein PPS auf. Jüngeres Alter und die geburtshilfliche Population gelten als die am besten belegten patientenbezogenen Risikofaktoren [1]. Neben patientenbezogenen Faktoren beeinflussen auch technische Aspekte das Risiko eines PPS. Hierzu zählen insbesondere ein größerer Nadeldurchmesser sowie schneidende Nadeln, während atraumatische Pencil-Point-Nadeln mit einem geringeren Risiko assoziiert sind [1, 4]. Im vorliegenden Fall wurde eine leitlinienkonforme 25-G-Pencil-Point-Nadel (Reganesth, Ermed AG, Schleitheim, Schweiz) mit Lidocain 1 % verwendet, und die Punktion verlief komplikationslos in einem Versuch. Trotzdem entwickelte die Patientin ein PPS, was die Bedeutung der nichtmodifizierbaren Risikofaktoren unterstreicht.

Die Pathophysiologie der Hörstörung beim PPS ist nicht vollständig geklärt. Diskutiert wird eine Übertragung des erniedrigten Liquordrucks über den Aquaeductus cochleae auf die Perilymphe des Innenohrs, wodurch ein negativer Druckgradient entsteht, der zu Veränderungen der intracochleären Mikromechanik führen kann. Dadurch könnten die Schwingungseigenschaften der Basilarmembran beeinflusst und bevorzugt tieffrequente Hörstörungen verursacht werden. Dieses Phänomen unterscheidet sich vermutlich von der Pathophysiologie des endolymphatischen Hydrops beim Morbus Menière, der heute nicht als reines Druckphänomen, sondern eher als Ausdruck komplexer mechanischer und elektrochemischer Veränderungen im Innenohr verstanden wird.

Die klassische Trias aus orthostatischen Kopfschmerzen, Tinnitus und Schwerhörigkeit wurde bereits in frühen Fallserien beschrieben [2]. Autophonie und Schwindel sind weitere typische Begleitsymptome der intrakraniellen Hypotension [3]. Die audiometrische Diagnostik mittels Reintonaudiogramm ist essenziell zur Objektivierung und Verlaufskontrolle der Hörstörung; weitere audiometrische Untersuchungen sind in der Regel nicht wegweisend. In einer systematischen Übersichtsarbeit zeigte sich bei acht von neun Patienten mit PPS eine Schwerhörigkeit im Tieftonbereich, bei sechs von neun Patienten war diese bilateral [2]. Eine vollständige Erholung des Hörvermögens wurde in sechs von neun Fällen beobachtet, wobei neuere Studien auf ein Risiko für chronische Hörstörungen hinweisen [2, 5].

Die konservative Therapie mit körperlicher Schonung, ausreichender Hydrierung, regelmäßiger Analgesie mit Paracetamol und nichtsteroidalen Antirheumatika sowie Koffeinzufuhr stellt die Erstlinientherapie bei mildem PPS dar [1]. Koffein kann in den ersten 24 h in Dosen bis zu 900 mg täglich verabreicht werden, bei stillenden Patientinnen sollte die Dosis auf maximal 300 mg täglich begrenzt werden. Im Gegensatz zum Hörsturz und zu anderen akuten Innenohrerkrankungen haben Kortikosteroide in der Therapie des PPS keinen Stellenwert. Besonders relevant ist die Empfehlung aktueller Leitlinien, bei Patienten mit schweren Symptomen (Hörverlust, Hirnnervenparesen) einen EBP als therapeutische Option zu erwägen [1]. Im vorliegenden Fall war aufgrund der raschen spontanen Besserung kein EBP erforderlich. Dennoch sollte bei persistierenden oder progredienten Ohrsymptomen die Indikation zum EBP großzügig gestellt werden, insbesondere da Langzeitstudien ein erhöhtes Risiko für chronische Hörbeeinträchtigung nach unbeabsichtigter Durapunktion zeigen (14 % vs. 2 % bei Kontrollen nach ≥ sechs Monaten; [5]).

Zusammenfassend zeigt der vorliegende Fall eine seltene, aber klinisch relevante cochleovestibuläre Manifestation des PPS nach Spinalanästhesie. Bei neu aufgetretener Tieftonschwerhörigkeit, Tinnitus, Autophonie oder Schwindel sollte gezielt nach einer vorausgegangenen Spinalanästhesie gefragt werden. Bei persistierenden oder progredienten Ohrsymptomen sollte interdisziplinär, insbesondere mit der Anästhesie, die Indikation zum EBP geprüft werden. Eine audiometrische Verlaufskontrolle ist sinnvoll, um die Regredienz zu dokumentieren und persistierende Hörstörungen frühzeitig zu erkennen.

## Fazit für die Praxis

- Das postpunktionelle Liquorverlustsyndrom (PPS) kann sich neben Kopfschmerzen mit bilateraler tieftonbetonter Schwerhörigkeit, Tinnitus und Autophonie manifestieren.
- Bei neu aufgetretener Hörstörung nach Spinalanästhesie sollte differenzialdiagnostisch an ein PPS gedacht, gezielt nach cochleovestibulären Symptomen gefragt und eine Reintonaudiometrie durchgeführt werden.
- Die Therapie ist primär konservativ; Kortikosteroide sind kontraindiziert, und bei persistierenden Symptomen sollte frühzeitig die Indikation zum epiduralen Blutpatch geprüft werden.

## Data Availability

Die im Rahmen dieses Fallberichts verwendeten Daten sind auf begründete Anfrage bei der korrespondierenden Autorin erhältlich.
